# Rotavirus A in Brazil: Molecular Epidemiology and Surveillance during 2018–2019

**DOI:** 10.3390/pathogens9070515

**Published:** 2020-06-27

**Authors:** Meylin Bautista Gutierrez, Alexandre Madi Fialho, Adriana Gonçalves Maranhão, Fábio Correia Malta, Juliana da Silva Ribeiro de Andrade, Rosane Maria Santos de Assis, Sérgio da Silva e Mouta, Marize Pereira Miagostovich, José Paulo Gagliardi Leite, Tulio Machado Fumian

**Affiliations:** Laboratory of Comparative and Environmental Virology, Oswaldo Cruz Institute, Oswaldo Cruz Foundation, Avenida Brasil 4365, Rio de Janeiro 21040-900, Brazil; meylin.gutierrez@ioc.fiocruz.br (M.B.G.); amfialho@ioc.fiocruz.br (A.M.F.); adriana.maranhao@ioc.fiocruz.br (A.G.M.); fabio.malta@ioc.fiocruz.br (F.C.M.); juliana@ioc.fiocruz.br (J.d.S.R.d.A.); rmsassis@ioc.fiocruz.br (R.M.S.d.A.); mouta@ioc.fiocruz.br (S.d.S.e.M.); marizepm@ioc.fiocruz.br (M.P.M.); jpgleite@ioc.fiocruz.br (J.P.G.L.)

**Keywords:** acute gastroenteritis, rotavirus A, incidence, genotyping, Brazil

## Abstract

Rotavirus A (RVA) vaccines succeeded in lowering the burden of acute gastroenteritis (AGE) worldwide, especially preventing severe disease and mortality. In 2019, Brazil completed 13 years of RVA vaccine implementation (Rotarix™) within the National Immunization Program (NIP), and as reported elsewhere, the use of Rotarix™ in the country has reduced childhood mortality and morbidity due to AGE. Even though both marketed vaccines are widely distributed, the surveillance of RVA causing AGE and the monitoring of circulating genotypes are important tools to keep tracking the epidemiological scenario and vaccines impact. Thus, our study investigated RVA epidemiological features, viral load and G and P genotypes circulation in children and adults presenting AGE symptoms in eleven states from three out of five regions in Brazil. By using TaqMan^®^-based one-step RT-qPCR, we investigated a total of 1536 stool samples collected from symptomatic inpatients, emergency department visits and outpatients from January 2018 to December 2019. G and P genotypes of RVA-positive samples were genetically characterized by multiplex RT-PCR or by nearly complete fragment sequencing. We detected RVA in 12% of samples, 10.5% in 2018 and 13.7% in 2019. A marked winter/spring seasonality was observed, especially in Southern Brazil. The most affected age group was children aged >24–60 months, with a positivity rate of 18.8% (*p* < 0.05). Evaluating shedding, we found a statistically lower RVA viral load in stool samples collected from children aged up to six months compared to the other age groups (*p* < 0.05). The genotype G3P[8] was the most prevalent during the two years (83.7% in 2018 and 65.5% in 2019), and nucleotide sequencing of some strains demonstrated that they belonged to the emergent equine-like G3P[8] genotype. The dominance of an emergent genotype causing AGE reinforces the need for continuous epidemiological surveillance to assess the impact of mass RVA immunization as well as to monitor the emergence of novel genotypes.

## 1. Introduction

Acute gastroenteritis (AGE) remains as a major cause of mortality in children under five years old worldwide [1,2]. Among the AGE-causing pathogens, rotavirus A (RVA) is one of the leading agents, responsible for approximately 200,000 deaths per year among children <5 years old in developing countries [3,4,5]. Regarding severe disease, RVA accounts for around 20% and 40% of all AGE-hospitalization in countries with and without RVA vaccines implemented, respectively [6,7]. Currently, four World Health Organization (WHO)-prequalified live-attenuated oral RVA vaccines are available internationally—Rotarix™, RotaTeq™, Rotavac™, and RotaSiil™—and over 100 countries have introduced one of these vaccines into their national immunization program [8] (https://www.who.int/immunization/diseases/rotavirus/en/).

Rotaviruses belong to the *Reoviridae* family, genus *Rotavirus*. While nine rotaviruses species have been described (A–I), RVA is by far the most important species infecting humans worldwide [9,10]. The non-enveloped triple-layered viral particle has 70–75 nm in diameter with 11 segmented double-stranded RNA (dsRNA) genes, encoding for six structural (VP1-VP4, VP6, VP7) and depending on the strain, five or six non-structural proteins (NSP1-NSP5 or NSP6) [11]. Genetically, RVA is classified into G- and P-types, based on nucleotide sequence of genomic segments coding VP7 and VP4 proteins (binary classification), and currently there have been described 36 G- and 51 P-types [12]. Although many G and P combination would be possible to emerge, a few genotypes (G1P[8], G2P[4], G3P[8], G4P[8], G9P[8], and G12P[8]) have prevailed worldwide causing the majority of RVA infections in children [13,14,15].

Brazil has implemented the Rotarix™ vaccine in the National Immunization Program (NIP) in March 2006, which led to a significant reduction of diarrhea-associated mortality and hospitalization [16,17,18]. Linhares et al. [19] demonstrated the higher effectiveness of Rotarix™ among Brazilian infants aged up to 12 months and decreasing in older children. Concerning the genotype distribution in Brazil after the introduction of Rotarix™, G2P[4] was by far the most prevalent genotype detected until 2010. From 2011 onwards, a gradual decrease in the prevalence of G2P[4] was observed, being replaced by G3, G9, and G12 harboring a P[8]-type [20,21,22,23]. Nevertheless, unusual RVA genotypes have been frequently detected, such as: G3[P6], G12[P6], G8P[4], and G8P[6] and more recently the equine-like G3P[8] [17,23,24]. Similarly, recent studies from other countries have reported the detection of rare RVA genotype combination [25,26,27,28].

It has been demonstrated that the distribution of RVA genotypes over the years is characterized by natural and cyclical genotype fluctuations [20,29,30]. However, the selective pressure due to mass RVA vaccination could favor specific G and P combinations [9,31]. Therefore, the new and dynamic epidemiological scenario reinforces the need to continuously document RVA prevalence in AGE cases, molecular epidemiology and the potential emergence of unusual genotypes.

Our study investigated RVA prevalence, features and the molecular characterization of G and P genotypes among patients with AGE from three regions (Southern, Southeastern and Northeastern) in Brazil, 2018–2019. RVA was detected and quantified by quantitative RT-PCR (RT-qPCR) from diarrheic stool samples received from eleven Brazilian states, and G and P genotypes were determined by multiplex one-step RT-PCR or sequencing.

## 2. Materials and Methods

### 2.1. Stool Collection and Ethics Statements

This study included stool samples that were collected between January 2018 and December 2019 from children and adults with symptoms of AGE, characterized as ≥three liquid/semi liquid evacuations in a 24 h period. Inpatients and outpatients diarrheic stool samples were collected from eleven states from three regions of Brazil: Southern, Southeastern, and Northeastern. Samples were systematically sent together with clinical-epidemiological records to the Regional Rotavirus Reference Laboratory–Laboratory of Comparative and Environmental Virology (RRRL–LVCA). The laboratory is part of the ongoing national network for AGE surveillance and coordinated by General Coordination of Public Health Laboratories, Brazilian Ministry of Health.

This study is approved by the Ethics Committee of the Oswaldo Cruz Foundation (FIOCRUZ), number CAAE: 94144918.3.0000.5248. The surveillance is performed through a hierarchical network in which samples are provided by medical request in hospitals and health centers, monitored by the Brazilian Unified Health System (SUS). Patients’ data were maintained anonymously and securely.

### 2.2. Viral RNA Extraction

Viral RNA was purified from 140 μL of clarified stool suspension (10% *w*/*v*) prepared with Tris-calcium buffer (pH = 7.2). Samples were subjected to an automatic nucleic acid extraction procedure using a QIAamp^®^ Viral RNA Mini kit (QIAGEN, CA, USA) and a QIAcube^®^ automated system (QIAGEN), according to the manufacturer’s instructions. RVA RNA was eluted in 60 µL of the elution buffer AVE. The isolated RNA was immediately stored at −80 °C until the molecular analysis. In each extraction procedure, RNAse/DNAse-free water was used as negative control.

### 2.3. RVA Detection and Quantification

RVA was detected and quantified by using a TaqMan^®^-based quantitative one step PCR (RT-qPCR) with primers and probe targeting the conserved NSP3 segment, according to Zeng et al. (2008). Briefly, RT-qPCR reactions were performed with 5 µL of the extracted RNA in a final volume of 25 µL using the SuperScript™ III Platinum™ One-Step qRT-PCR Kit (ThermoFisher Scientific, Invitrogen Division, Carlsbad, CA, USA) in the Applied Biosystems^®^ 7500 Real-Time PCR System (Applied Biosystems, Foster City, CA, USA). NSP3 primers and probe final concentrations used were 0.8 and 0.5 µM, respectively. The thermal cycling conditions were carried out as follows: RT step at 55 °C for 30 min, an initial denaturation step at 95 °C for 10 min and 40 cycles of PCR amplification at 95 °C for 15 s and 60 °C for 1 min. Samples that crossed the threshold line showing a characteristic sigmoid curve were regarded as positive. All runs included negative and non-template controls, and a standard curve with serial dilutions (10^6^–10^1^) of double-stranded DNA fragments (gBlock^®^ Gene Fragment, Integrated DNA Technologies, Iowa, USA) containing the RVA NSP3 target region to ensure the correct interpretation of the results throughout the study. RVA viral loads were expressed as genome copies per gram (GC/g) of stool.

### 2.4. Genotyping and Sequencing

RVA-positive samples obtained by RT-qPCR were G- and P-genotyped using a one-step multiplex RT-PCR. The reactions were performed using the Qiagen One Step RT-PCR kit (Qiagen), using forward conserved primers VP7uF or VP4uF and specific reverse primers for G types G1, G2, G3, G4, G9, and G12, or P types P[4], P[6], P[8], P[9], and P[10] as recommended by the Centers for Disease Control and Prevention, USA. The G- and P-genotypes were assigned based on different amplicon sizes [base pairs (bp)] using agarose gel analysis. Sanger sequencing was also used to characterize the nucleotide (nt) sequence of specific strains, such as non-typeable samples or the equine-like G3, using consensus primers directed to the conserved regions within the VP4 and VP7 genes. The amplicons fragments of 876 bp and 881 bp for VP4 and VP7, respectively, were purified using the ExoSAP clean-up kit (ThermoFisher Scientific) and sent to the FIOCRUZ Institutional Platform for DNA sequencing (PDTIS). All primers used for RVA genotyping were based on previously studies [32,33,34].

### 2.5. Phylogenetic Analysis

Chromatogram analysis and consensus sequences were obtained using Geneious Prime (Biomatters Ltd., Auckland, New Zealand). RVA genotypes were confirmed in terms of closest homology sequence using Basic Local Alignment Search Tool (BLAST). Phylogenetic trees were constructed using the maximum likelihood method and the Kimura two-parameter model (2000 bootstrap replications for branch support) in MEGA X v. 10.1.7 [35], with RVA reference sequences obtained from the National Center for Biotechnology Information (NCBI) database. Nucleotide sequences obtained from clinical samples were submitted to NCBI GenBank (accession numbers: MT386419 to MT386453).

### 2.6. Statistical Analysis

Statistical analyses were performed using GraphPad Prism software v. 8.4.1 (GraphPad Software, San Diego, CA, USA). As appropriate, Mann–Whitney U test, Chi-squared or Fisher test was used to assess significant difference between RVA detection rates, years of collecting samples and age groups, as well as to compare RVA viral load according to different age groups. A *p* value < 0.05 was considered to be statistically significant.

## 3. Results

### 3.1. Rotavirus A Epidemiology

**Figure 1:**
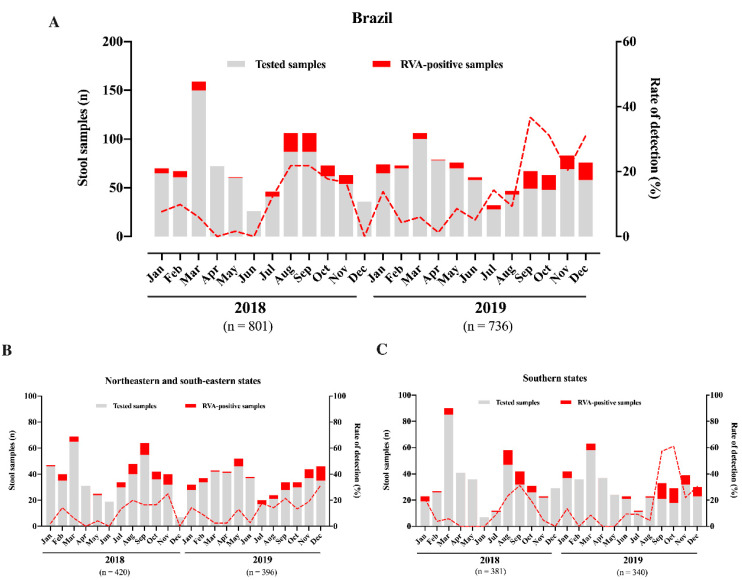
Monthly distribution of tested acute gastroenteritis samples, rotavirus A (RVA)-positive samples and RVA detection rates in Brazil (**A**), Northeastern and South-eastern states (**B**), and Southern states (**C**), during 2018–2019.

During the two-year period of this study (2018–2019), a total of 1536 stool samples were collected from symptomatic inpatients with AGE (1161 and 375 from children and adults, respectively). Overall, we detected RVA in 12% of samples (n = 185), 10.5% in 2018 and 13.7% in 2019. We observed a slight increase in RVA incidence in 2019, but without statistical significance (*p* = 0.053). Except for three months in 2018 (April, June, and December), RVA circulated year-round, with monthly detection rates varying from 1.6% to 36.7% in May 2018 and September 2019, respectively (Figure 1A). In relation to seasonal patterns, we observed higher RVA circulation during winter/spring months, especially marked in Southern region states (Figure 1B,C), whilst RVA detections were lowest in autumn months.

**Figure 2:**
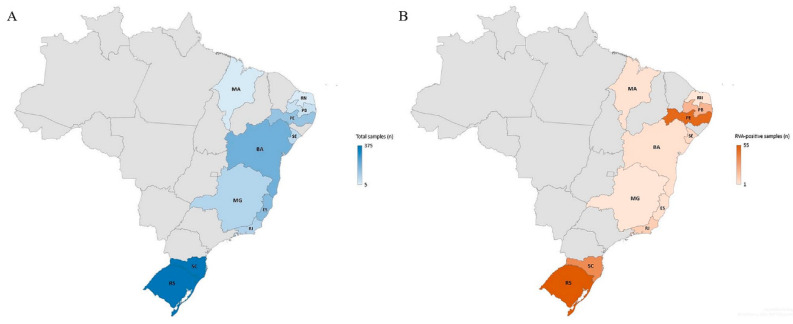
Map of Brazil highlighting the eleven states with sentinel surveillance service attended by the Rotavirus Regional Reference Laboratory, IOC, FIOCRUZ. Number of tested samples (**A**) and number of RVA-positive samples (**B**).

In regard to regional analysis, higher RVA prevalence was observed in the Northeast region (18.7%) compared to Southeastern and Southern regions (3.4% and 12.5%, respectively). Comparing the two year of the study, RVA detection rates were higher in 2019 for the three regions, but only with statistical significance in Southeastern region (*p* = 0.022). Table 1 shows detailed analysis by regions and states. It is interesting to note that the two states of Southern region (Santa Catarina and Rio Grande do Sul) accounted for almost half of the AGE cases and RVA-positive samples (Figure 2).

**Figure 3:**
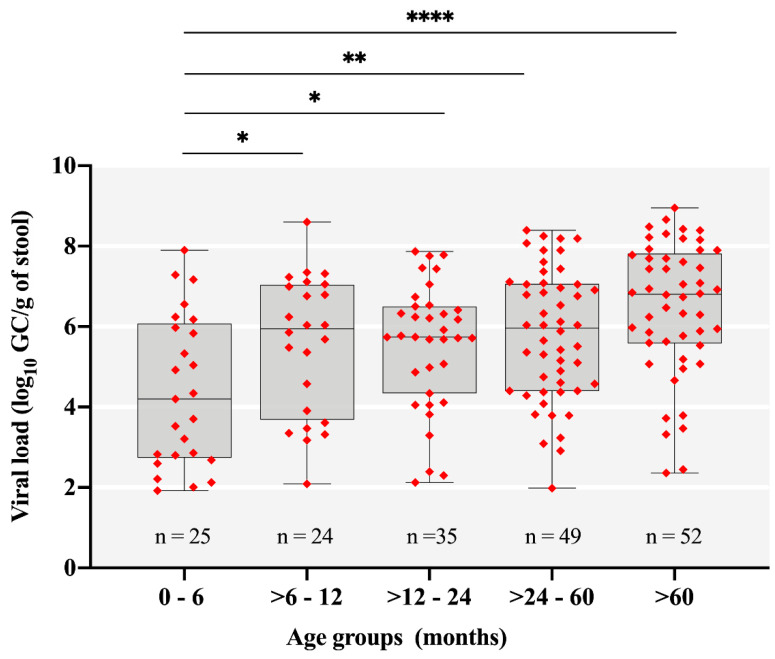
Rotavirus A (RVA) viral load expressed as log_10_ genome copies per gram of stool (log_10_ GC/g) among different age groups in Brazil, 2018–2019. Box-and-whisker plots show the first and third quartiles (equivalent to the 5th and 95th percentiles), the median (the horizontal line in the box), and range of log_10_ GC/g values. * *p* ≤ 0.05; ** *p* ≤ 0.01; **** *p* ≤ 0.0001.

Most of stool samples received were from children less than five years old, representing 72.1% (1108/1536) of the AGE cases. RVA detection rate was significantly higher among children aged between 24 and 60 months (18.8%) compared to the other age groups, where detection rates varied from 9.3% to 12.1% (Table 2). We also analyzed RVA viral load (GC/g of stool) among different age groups. The median values of RVA viral loads varied from 4.2 to 6.8 log_10_ ⁠GC/g among the different age groups. RVA-positive samples showed viral load values statistically lower in AGE cases among children ≤6 months compared to older patients (*p* < 0.05) (Figure 3).

### 3.2. RVA Genotyping

**Figure 4:**
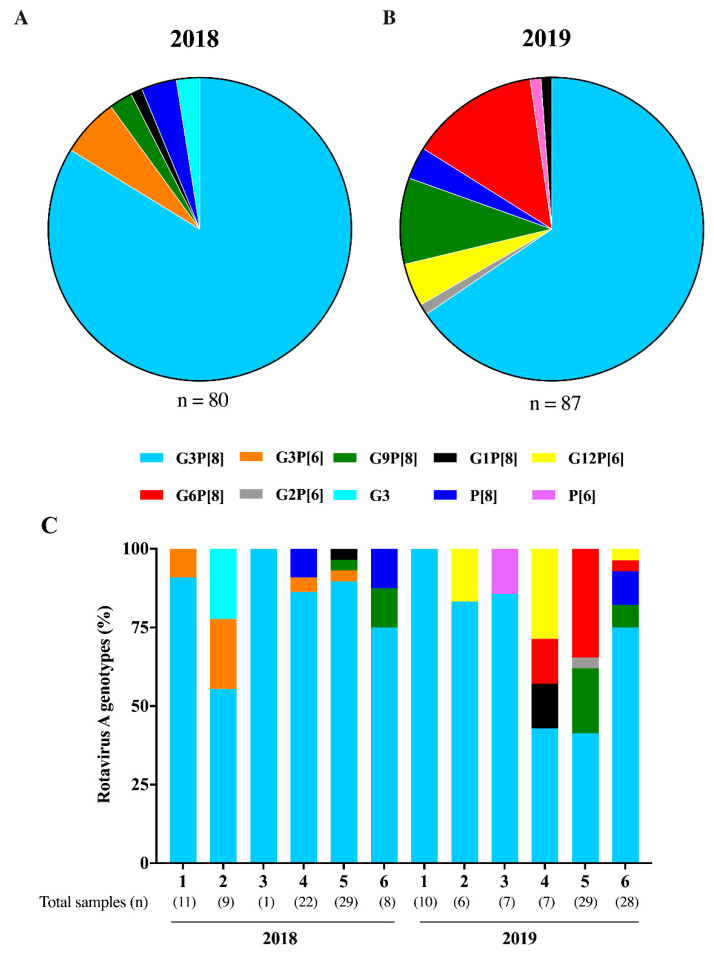
Rotavirus A (RVA) genotypes distribution in Brazil, 2018 (**A**) and 2019 (**B**). Bi-monthly genotypes circulation during the two-year of study (**C**).

A total of 186 RVA-positive samples were subjected to G and P genotyping by one-step multiplex RT-PCR. From these, 167 samples (89%) were successfully genotyped; 80 from 2018 and 87 from 2019. We characterized seven different RVA genotypes circulating during this study: G3P[8], G3P[6], G9P[8], G1P[8], G2P[6], G12P[6], and G6P[8]. G3P[8] was detected year-round and was by far, the most prevalent genotype, accounting for 83.8% (n = 67) of genotyped samples in 2018 and 65.5% (n = 57) in 2019 (Figure 4). Two other usual RVA genotypes were detected, but in lower prevalence—G1P[8] detected in one sample in 2018 and 2019, and G9P[8] detected in two and eight samples from 2018 and 2019, respectively. We also detected unusual G/P combinations, especially in 2019, as follows: G3P[6] in 6.3% of samples from 2018; G6P[8], G12P[6], and G2P[6] in 13.8%, 4.6% and 1.2% of samples from 2019 (Figure 4). G or P non-typed (NT) samples (GNTP8, GNTP6, and G3P[NT]) accounted for 5.4% of samples, and were represented mostly by samples with low RVA viral load (high Ct values).

**Figure 5:**
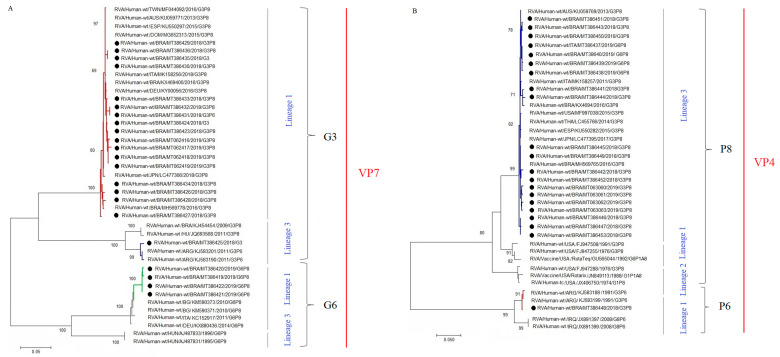
Phylogenetic analyses based on VP7 and VP4 nucleotide (nt) sequences of circulating Brazilian rotavirus strains. Strains obtained in this study are marked with a black filled circle and names contain the register number, state, and collection date (M/Y). Reference strains were downloaded from GenBank and labeled with their accession number followed by country, register number, year, and genotype. Neighbor-joining phylogenetic trees of VP4 (**A**) and VP7 (**B**) were constructed with MEGA X software and bootstrap tests (2000 replicates) based on the Kimura two-parameter model. Bootstrap values above 70% are given at branch nodes.

In addition to RT-PCR genotyping, we sequenced some of the RVA-positive samples in order to get detailed information of the circulating strains and their respective lineages. We successfully obtained 22 and 21 consensus sequences of VP7 and VP4 genes, respectively. Phylogenetic analysis of the VP7 gene confirmed the characterization of Brazilian strains belonging to G3 and G6. Eighteen G3 strains from both years and from the three Brazilian regions were sequenced. From these, 94.4% (n = 17) of sequences clustered within the lineage 1, represented by equine-like G3P[8] strains. Our sequences were genetically related to previously detected equine-like G3P[8] strains from Brazil (KX469400) and other countries, such as Germany (KY000546), Slovakia (MN203563), Dominican Republic (MG652313), and Japan (LC47366). One G3 sequence clustered within lineage 3 that comprises the Wa-like G3P[8] group. The Brazilian Wa-like G3 sequence was closely related to strains from Brazil (KJ454454), Argentina (KJ583190 and KJ583201) and Hungary (JQ693568), with nt similarity varying from 98.4 to 99.8% (Figure 5A). The four G6 strains sequenced in our study, harboring a P[8]-type, clustered within lineage 1 showing moderate nt identity (97.8–98.1%) with G6P[8] strains detected in Bulgaria (KM590371 and KM590373) and with G6P[9] strains from Germany (KX880436) and Italy (KC152917). None of our G6 sequences clustered within the G6 lineage 3, that comprises human-bovine reassortant strains (Figure 5A).

Phylogenetic analysis of 21 sequences of VP4 gene, demonstrated that, except for one, all P[8] Brazilian strains harboring two different G-types (G3 and G6) grouped into lineage 3. The 20 strains were closely related (99.2–99.6% of nt similarity) to P[8]-3 Brazilian strains isolated in 2016 (KX469415 and MH569765) and strains from other countries, such as USA (MF997038), Japan (LC477395), Spain (KU550282), Australia (KU059769), and Italy (MK158257). One strain was characterized into P[6] lineage 1, and was closely related (99.5–99.7% of nt similarity) to strains detected in Argentina (KJ583199), Iraq (JX891397), and China (MG78835) (Figure 5B).

## 4. Discussion

In this study, we provide laboratory-based RVA national surveillance in eleven states from three regions in Brazil, during 2018–2019. We tested 1536 AGE stool samples and found an overall RVA-positivity of 12%. RVA detection rates were higher during winter/spring months and among children aged 24–60 months. By far, G3P[8] was the most frequently detected genotype, and showed a year-round circulation.

Despite the development of vaccines, RVA are still a major cause of severe AGE in infants worldwide [5]. Here, we detected RVA in 10.5% and 13.7% of samples from 2018 and 2019, respectively. In Brazil, after Rotarix™ implementation, different studies have investigated RVA circulation among AGE cases. A study from the Enteric Diseases Laboratory at Adolfo Lutz Institute, one of the three Brazilian Reference Laboratory for RVA surveillance, reported annual RVA prevalence varying from 9.9% to 25.3% during 2013–2017, with AGE samples from five states in the Midwestern and part of the Southeastern and Southern regions [24]. A previous study from our group demonstrated an overall RVA positivity of 20.8% among children up to 12 years old between 2006 and 2017, with annual detection rates varying between 5% to 35% [20]. Studies conducted at Evandro Chagas Institute, the national and regional reference center for RVA surveillance in Northern Brazil, demonstrated RVA positivity rates of 33% in samples from six states from North Brazil, 2011–2012 [23], and 24.2% in samples collected between June 2012 and June 2015 [36]. However, it is worth mentioning that both studies involved children hospitalized for severe AGE. In Argentina, RVA positivity decreased from 26.8% to 13.6% comparing the pre- and post-vaccination periods [37]. Other studies performed elsewhere have described RVA detection rates varying from 8.4% to 23.2% [38,39,40,41,42].

RVA seasonality has been well defined, especially for temperate climate countries, where RVA peaks during dry and cold months. In tropical areas, RVA circulates year-round without marked peaks of infections [43,44]. In Brazil, we observed a year-round RVA circulation without marked seasonality, but high detections rates of RVA was observed during winter/spring months, in agreement with other studies [42,45]. RVA highest detection rate was observed in September 2019 (36.7%) in line with findings observed over a 21-year period in Brazil [20], and also with Luchs et al. [46] that demonstrated the peak of RV incidence in September during a five-year RVA surveillance study (2007–2012) in Brazil. As a continental-size country, we analyzed separately, RVA circulation in Southern states in comparison with Southeastern and Northeastern states (Figure 1B,C). We observed more clear peaks of RVA infections in winter/spring months (June 21st to December 20th) in Southern states (Rio Grande do Sul—RS, and Santa Catarina—SC) compared to Southeastern and Northeastern Brazil. This could be explained as both RS and SC states are in a subtropical area, characterized by different climate pattern compared to the other states. A three-year study conducted in Vietnam to access RVA epidemiology in AGE cases also demonstrated varied seasonally positivity, with different RVA-detection peaks among the three regions analyzed—North, Central, and South [47]. The fact that RVA usually peaks in September in Brazil, observed here and by others [20,46], is an important information to authorities to prepare strategies to reduce AGE impacts in the health system.

Regarding RVA infections among different age groups, we observed a significantly high positivity rate among children aged >24 and 60 months compared to other age groups. This shifting in the age of children more affected by RVA illness (older children) has been observed, especially in countries that have introduced RVA mass vaccination. Our data are consistent with previous findings reported from Brazil [20] and the USA [48,49]. In contrast, countries where RVA vaccines are yet to be introduced into national immunization programs, have reported the majority of RVA positive children (~90%) within the first 2 years of life [44,47]. By analyzing RVA shedding among the age groups, we found a statistically lower viral load among children less than six months (Figure 3). We believe that this lower viral load could be mostly explained by the passive protection mediated by breast milk maternal antibodies [50], but also by the higher effectiveness and prompt immune response generated by Rotarix™ after the oral doses administered at the age of 2 and 4 months [17]. However, this second hypothesis alone could not explain the high viral load among children aged >6 and 12 months. In addition, high Ct values could indicate less severe disease [51]. In that study, authors demonstrated that the severity of diarrhea, determined by the Vesikari score, was significantly and negatively associated with Ct values of children stool samples.

Regarding RVA genotype characterization, we successfully identified G- and P-types in 89% of positive samples, by one-step multiplex RT-PCR and sequencing. By far, G3P[8] was the most prevalent genotype in both years. The phylogenetic analysis of the VP7 gene revealed that the majority of the Brazilian strains sequenced (94%) belong to the equine-like G3 genotype (G3-1). Moreover, all the P[8] strains sequenced clustered within the P[8]-3 lineage. This P[8]-3, harboring a G12-type, was the dominant strain in Brazil in 2014, detected in 75% of genotyped samples [52].

Emergent equine-like DS-1-like G3P[8] RVA strains were firstly identified in children with AGE in Australia in 2013 [53]. From 2013 onwards, the equine-like G3P[8] DS-1-like genotype has spread and become endemic worldwide [54,55,56,57,58,59,60]. In Brazil, the first evidence of the circulation of equine-like G3P[8] date from 2015, when Luchs et al. [24] detected the reassortant RVA strain in a touristic city of Southern Brazil, Foz do Iguaçu, that borders Argentina and Paraguay. Subsequently, these novel viruses quickly spread to other states in Brazil, being the most prevalent genotype in 2017 (66.2%). The occurrence of DS-1-like G3P[8] RVA strains was also reported in Amazon region, Northern Brazil in 2016 [60]. In the previous study from our group, we demonstrated the increase of G3P[8] from 2015, peaking in the last year of the study—2017. However, it was not investigated whether they belonged to the DS-1-like RVA group [20]. More recently, countries such as Australia, Italy and Pakistan, have demonstrated the high prevalence of the emergent equine-like G3P[8] genotype [42,45,61].

Atypical genotypes G3P[6], G6P[8], G2P[6], and G12P[6] were also detected as minor genotypes in our study. The phylogenetic analysis of the VP7 gene demonstrated that Brazilian G6-1 strains were closely related to strains circulating in Bulgaria and Italy [62,63]. The genotype G12P[6] characterized in our study has been frequently detected in Nepal, with detection rates of 46.4% in 2013 and 36% in 2014, among AGE cases in children less than five years of age [64,65]. Unexpectedly, we did not detected the former dominant G2P[4] genotype. In Brazil, after Rotarix™ implementation in March 2006, this genotype has been the most frequently detected until 2015 [66], however, the recently low prevalence of G2P[4] viruses could be explained a cyclical pattern of circulation along with the herd induced homotypic immunity and depletion of the susceptible population [20].

A major strength of our study is that we included data from eleven states, representing around 100 million inhabitants (almost half of Brazilian population). Albeit, this could be considered as a major limitation as well, since the variability in reporting and collecting AGE cases by states generates surveillance biases. Another limitation is that important RVA genes, such as VP6 and NSP4, were not characterized. Nevertheless, future studies approaching a more complete genetic characterization of G3P[8] strains, as well as unusual genotypes detected here (G3P[6] and G12P[6]) will be performed, in order to monitor RVA genotypes spread and evolution over time.

In conclusion, we found a 12% of RVA-positivity in AGE cases from Brazil, and according to global trends, the equine-like G3P[8] was the dominant genotype in 2018 and 2019. The constant shifting of RVA genotypes circulation and the potential emergence of unusual/reassortant strains reinforces the importance and the need for continuous country-based epidemiological and molecular surveillance programs.

## Figures and Tables

**Table 1:** Number of tested and rotavirus-positive fecal samples through laboratory-based surveillance by region and state in Brazil during 2018 and 2019.

Region/State	No. of Fecal Samples: Positive/Tested (%)			*p*-Value (Chi-Square Test)
	Total	2018	2019	
**Southeast**	14/381 (3.7)	2/168 (1.2)	12/213 (5.6)	0.022
Espírito Santo		1/56	2/101	
Minas Gerais		1/75	-	
Rio de Janeiro		-	10/79	
**Northeast**	81/434 (18.7)	44/252 (17.5)	37/182 (20.3)	0.452
Bahia		1/98	2/95	
Maranhão		1/8	1/1	
Paraíba		20/37	-	
Pernambuco		19/68	30/61	
Rio Grande do Norte		-	1/5	
Sergipe		3/41	3/20	
**South**	90/720 (12.5)	39/381 (10.2)	51/340 (15)	0.053
Rio Grande do Sul		16/168	38/181	
Santa Catarina		23/213	13/159	

**Table 2:** Number of tested and rotavirus-positive fecal samples through laboratory-based surveillance by age group in Brazil during 2018–2019.

Age Group (Months)	No. of Fecal Samples: Positive/Tested (%)			*p*-Value * (Chi-Square Test)
	2018	2019	Total	
0–6	16/122 (13.1)	9/101 (8.9)	25/223 (11.2)	0.0153
>6–12	10/133 (7.5)	14/116 (12)	24/249 (9.6)	0.0021
>12–24	17/203 (8.3)	18/173 (10.4)	35/376 (9.3)	0.0003
>24–60	26/141 (18.4)	23/119 (19.3)	49/260 (18.8)	-
>60	16/202 (7.9)	36/227 (15.8)	52/428 (12.1)	0.0109

* *p*-values were calculated between the age group of >24–60 and each other. All other combinations were not statistically different.
